# Surgical Treatment of Pruritus Vulvæ

**Published:** 1886-02

**Authors:** 


### Surgical Treatment of Pruritus Vulvæ.

Pruritus vulvæ depends often upon continued vaginal or uterine discharges. These discharges bathe continually the mucous membrane of the vulva and by their presence produce an irritation which causes the pruritus. When treatment is early directed to these discharges, their stoppage often cures the disease; when, however, they have lasted a long time, the effect on the mucous membrane is such that even after the discharges have been stopped, the puritus will persist more or less tenaciously. Kustner (*Centralbl. für Gynæk.*) suggests a new method of treatment with which he has been successful in four cases. He excises the mucous membrane at the seat of trouble, and reunites the borders of the cut surface by means of sutures. Cicatrization is rapid and the pruritus disappear simmediately after the operation. According to the author, in the majority of cases, local measures are necessary, and nothing short of resection of the nerve endings in the mucous membrane is capable of affording relief. This resection is accomplished by the operation which Kustner has done, by dissecting away the mucous membrane at the seat of the pruritus. (*Bulletin Général de Thérapeutique,* 15 Octobre, 1885.)

---

Dr. S. J. Smith, in *Physicians' and Surgeons' Investigator,* says: "The hair is essentially the same structure as the epidermis. It may be found on nearly every part of the surface of the body, excepting the palms of the hands and soles of the feet.

"In cases of ordinary madesia, or falling of the hair, Dr. E. Wilson recommends strong liquor ammonia, almond oil, chloroform, of each one part; alcohol or spirits of rosemary, five parts; essential oil of lemon, one drachm. Apply daily, freely, and after thorough friction with a hair-brush. As a hair tonic for internal use, he thinks 'arsenic bears the palm.' In loss of the hair, Dr. Shoemaker recommends 'tinc. ignatia drops ten, or sulphurous acid ʒss., three times a day, with a bitter tonic.' The following is recommended as a hair restorative:

| | | |
|---|---|---|
| ℞ | Bay Rum, - - - - | |
| | Glycerine, - - - | aa. ʒii. |
| | Tinc. Cantharides, - - - | |
| | Tinc. Capsicum, - - - | aa. ʒi. |
| | Quinia Sulph., - - - | grs. x. |
| | Aqua, - - - - | ℥iv. M. |

"Use daily in place of oil for a dressing.

"In cases of alopecia which have lasted so long that the hair bulbs have become atrophied, nothing will restore the hair on those spots. Our endeavors must be directed to saving what remains. A prognosis favorable to the restoration of the hair must, therefore, be given with caution. After cleansing of the scalp with soap and water, direct this lotion to be used freely:

| | | | |
|---|---|---|---|
| ℞ | Bay Rum, - - - - | | Oij. |
| | Tinc. Lobelia, - - - | | |
| | Tinc. Sanguinaria, - - - | aa. | ℥i. |
| | Tinc. Cantharides, - - | | ℥ii. |
| | Boracis, - - - - | | ℥ss. M." |

